# Trends and Outcomes of Hospitalizations Due to Hemolytic Uremic Syndrome: A National Perspective

**DOI:** 10.7759/cureus.32315

**Published:** 2022-12-08

**Authors:** Elvina Lingas, Jiya Mulayamkuzhiyil Saju, Mohammed Ali Abdulqader, Deeke Yolmo, Madiha Shaikh, Padmanayayakege Chamithra Dilshani Rupasinghe, Achint A Patel

**Affiliations:** 1 Internal Medicine, Presbyterian Hospital, Albuquerque, USA; 2 Internal Medicine/General Surgery, Government Medical College, Trivandrum, IND; 3 Internal Medicine/Pediatrics, Geisinger Medical Center, Danville, USA; 4 Internal Medicine/ENT, Jawaharlal Institute of Postgraduate Medical Education & Research, Puducherry, IND; 5 Pathology, Indira Gandhi Government Medical College & Hospital, Nagpur, IND; 6 Internal Medicine, Manipal College of Medical Sciences, Pokhara, NPL; 7 Internal Medicine, HCA Florida Oak Hill Hospital/USF (University of South Florida) Health Morsani College of Medicine, Brooksville, USA

**Keywords:** nationwide inpatient sample, hemolytic uremic syndrome hospitalization, in hospital mortality, outcome analysis, trend analysis, hemolytic uremic syndrome

## Abstract

Background: Hemolytic uremic syndrome (HUS) is a rare but challenging disease with varying degrees of mortality and prognosis. We aim to evaluate the trends and outcomes of hospitalizations due to HUS by utilizing a large population-based dataset.

Methods: We derived a study cohort from the Nationwide Inpatient Sample (NIS) for the years 2007-2018. Our primary outcomes were in-hospital mortality, discharge disposition, and predictors of poor outcomes. We then utilized the Cochran Armitage trend test and multivariable survey logistic regression models to analyze the trends, outcomes, and predictors.

Results: A total of 8043 hospitalizations ranging from age zero to above 65 years of age occurred due to HUS from 2007-2018. The number of hospitalizations with HUS increased steadily from 528 in 2007 to 800 in 2013, but afterwards, we noticed a steady decline to 620 in 2018. Additionally, trends of in-hospital mortality slowly increased over the study period but we noticed a decline in the rate of discharge to skilled nursing facilities (SNFs). Furthermore, in multivariable regression analysis, predictors of increased mortality in hospitalized HUS patients were advanced age (95%CI: 1.221-1.686; p-value <0.0001) and requirement for dialysis (95%CI: 1.141-4.167; p-value: <0.0001). Advanced age >65 years (OR: 2.599, 95%CI: 1.406-4.803; p-value: 0.0023), as well as comorbidities such as diabetes mellitus and pulmonary circulatory diseases, which are under vascular events (OR: 1.467, 95%CI:1.075-2.000; p-value: 0.0156), were shown to have a higher rate of discharge to SNFs. Moreover, patients needing intravenous immunoglobulin (IVIG) and plasmapheresis had high odds of discharge to SNFs ((OR: 1.99, 95%CI: 1.307-3.03; p-value: 0.0013) and (OR: 5.509, 95%CI: 2.807- 10.809; p-value <0.0001), respectively), as well as smaller hospital bed size and hospital type (OR: 1.849, 95%CI: 1.142-2.993; p-value: 0.012).

Conclusion: In this national representative study, we observed a total decrease in hospitalizations as well as discharge to SNFs; however we saw an increase in inpatient mortality. We also identified multiple predictors significantly associated with increased mortality, some of which are potentially modifiable and can be points of interest for future studies.

## Introduction

Hemolytic uremic syndrome (HUS) is a clinical entity that is characterized by concurrent episodes of microangiopathic hemolytic anemia, thrombocytopenia, and acute kidney injury (AKI). It is a clinical syndrome that mostly presents after gastrointestinal viral infection and is associated with autoimmune reactions [1]. It occurs most commonly in children although it also may happen in adults. Atypical HUS is most used in describing presentation in adults, although it can also happen in children [2]. The yearly incidence of typical HUS is three cases per 100,000 [3]. In the United States, typical HUS occurs most frequently in rural communities during the summer and fall months [3]. Atypical HUS occurs more sporadically, with an estimated incidence of 10 cases per 1,000,000 in the United States, and about seven per 1,000,000 in Europe [4]. It has been shown that the incidence of HUS varies according to country and climate and it tends to show a higher trend in colder countries [5].

There is limited data when it comes to hospitalization trends in adults. In a study cohort of Nationwide Inpatient Sample (NIS) for the years 2008-2017, it was demonstrated that among hospitalized patients, more than half were children and followed by 24% of adults ranging from 18 to 65 years of age [6]. The mortality rate was calculated to be around 3%, which is consistent with other studies that documented mortality of less than 5% in children [7,8], although several other studies have documented varying degrees of mortality specifically in adults [9].

Management is usually mainly supportive and targeted to the underlying cause, which is often unclear. While plasma exchange has been shown to be beneficial in thrombotic thrombocytopenic purpura (TTP), its role in HUS in adults remains unclear. There have been no randomized controlled trials that evaluated plasma exchanged in HUS. In an Italian observational study that included 273 patients with HUS who received plasma exchange, over half (55-80%) improved [10].

Anti-complement monoclonal antibodies such as eculizumab have also been used to treat HUS; however, the recommendation is based on observational studies as supporting evidence since there has been no randomized controlled trial available [11]. A systematic review of two uncontrolled prospective studies and one small uncontrolled retrospective study showed that eculizumab is effective in reducing thrombotic microangiopathy (TMA) activity as measured by TMA-free event and normalization of platelet count [11]. The time between HUS and treatment initiation determines outcomes of atypical HUS, such as an improvement in glomerular filtration rate (GFR) [12]. A three-to-six-month treatment is recommended due to the chance of late renal recovery [13]. Pre and post-operative eculizumab has also increased the success rate of kidney transplantation in children with HUS [14]. Long-term data on eculizumab is still limited; although this observational study showed that the mean GFR of participants improved and remained stable up to six years on eculizumab while there is a trend of worsening GFR in the counterpart group [15]. 

Different studies have documented different factors in children that could affect outcomes including age, duration of hospitalization, leukocyte count, hemoconcentration, and need for dialysis [14,16]. In adults, being HIV positive, having underlying kidney issues, and need for dialysis seem to worsen prognosis [9]. There is still limited data on hospitalization trends in general, including in-hospital mortality and predictors of poor outcomes.

This study aims to describe hospitalization trends, patient characteristics, and in-hospital outcomes of HUS using a national database.

## Materials and methods

Study design

The study population was obtained from the NIS 2007-2018 database. The study aimed on obtaining hospitalization trends, characteristics, and outcomes of HUS among hospitalized cases.

Inclusion and exclusion criteria

Our study was extracted from the NIS of the Agency for Healthcare Research and Quality (AHRQ) and Healthcare Cost and Utilization Project (HCUP). NIS is considered one of the largest available databases of inpatient hospitalizations from United States hospitals. Only hospitalized patients with HUS were included. 

Statistical analysis

Descriptive statistics were performed to present the baseline sociodemographic, comorbidities, and hospital-level characteristics of HUS hospitalizations. Proportions and means were used to summarize categorical and continuous variables, respectively. The exposure variable was the calendar year of HUS hospitalizations. The outcomes of interest were trends in HUS hospitalizations, mortality, discharge disposition, length of stay (LOS), and predictors of poor outcomes.

## Results

Temporal trends of HUS hospitalizations

**Figure 1:**
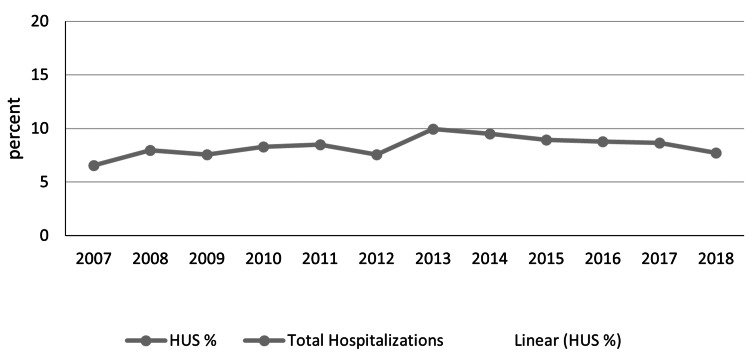
Temporal trend of hemolytic uremic syndrome HUS: hemolytic uremic syndrome

In trend analysis, we observed a steady incline in yearly hospitalization related to HUS from 528,216 in 2007 to 800,000 in 2013; however, we subsequently saw a decline in hospitalization from 2013 to 2018 (Figure 1). The proportion of patients being hospitalized with HUS is still higher in 2018 at 7.71% compared to 6.57% in 2007.

Temporal trends of HUS hospitalizations by demographics

**Figure 2:**
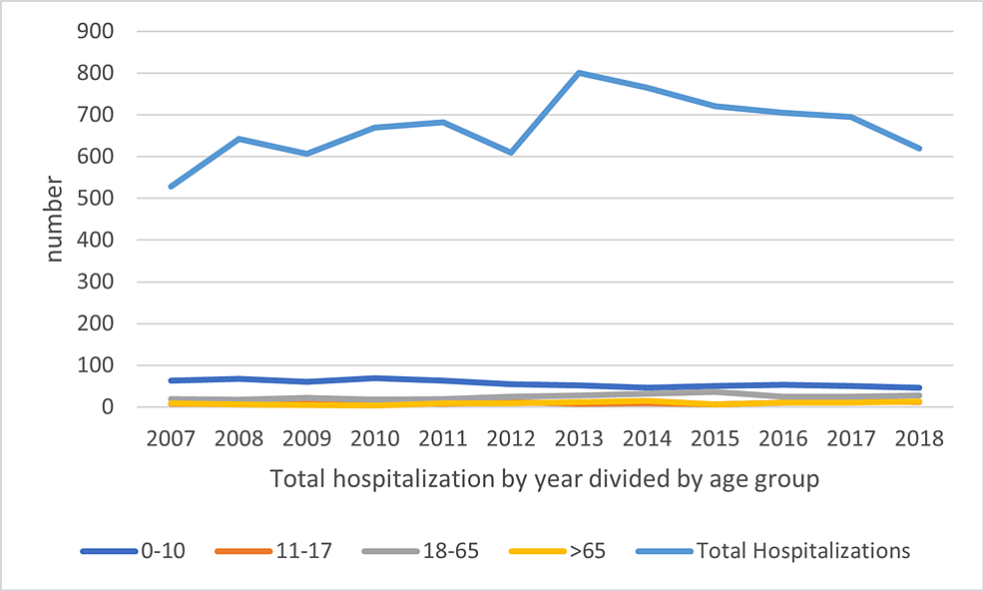
Temporal trend by age

We observed a steady decline in hospitalization rate in the age group of 0-10 years from up to 63.8% hospitalized HUS patients in 2007 to 46.77% in 2018 (Figure 2). However, as Figure 1 demonstrated, we observed a steady incline in hospitalization rates in other age groups. 

**Figure 3:**
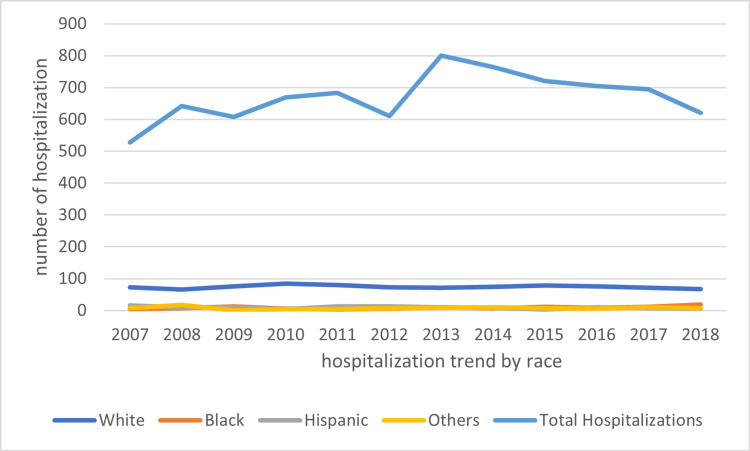
Trend of hospitalization by race

Most hospitalized HUS patients are White, which consistently makes up more than 50% of all hospitalized HUS cases. We observed a steady decline in hospitalization in White patients from 73.08% in 2007 to 67.83% in 2018 (Figure 3). A similar trend is observed in Hispanic groups whose hospitalization rate declined from 15.55% in 2007 to 6.09% in 2018. By contrast, we observed a significant incline in Black patients from 4.19% to 19.13%. There is also a steady incline in female patients from 60.67% to 63.71% whereas we saw a decline in male patients from 39.33% to 36.29%

Characteristics of hospitalized HUS patients

Most of the patients admitted with HUS in our study were in the 0-10 age group (56.2%), followed by the 18-65 age group (24.7%). Patients were mostly females (59.5%), White (74.4%), and had comorbidities like coagulopathy (56%), hypertension (45.6%), chronic pulmonary disease (7.2%), weight loss (6.3%), congestive heart failure (5.5%), diabetes mellitus without complications (4.5%), and hypothyroidism (3.7%). There was no evident trend between median household income and HUS hospitalizations. Private insurance including Health Maintenance Organization (HMO) paid for 53.5% of the HUS hospitalizations while Medicare/Medicaid paid for 38.1% of the hospitalizations. Patients were admitted more to large-bed-sized hospitals (64.3%) and 83.1% of the admissions were to teaching hospitals; 58.6% of admissions came as transfers from other hospitals or skilled nursing facilities (SNFs) while 41.4% were emergency department admissions. The majority of the admissions were on weekdays (77.2%) and 90.6% of all hospitalizations occurred on an emergency or urgent basis. The southern region of the United States recorded the highest percentage of HUS hospitalizations at 33.3%. 

Treatment/complication of hospitalized HUS patients

Of patients with HUS, 18.1% required plasmapheresis, and treatment with plasmapheresis demonstrated a stable trend over the years from 2007 (17.76%) to 2018 (15.32%). Overall, 5.9% of patients had vascular events, which depicted an increasing trend from 6.66% in 2007 to 9.68% in 2018. AKI occurred in 66.9% of HUS patients, which showed a rise in AKI events in the last five years of our study, i.e., 77.42% in 2018 compared to 54.82% in 2007; 29.8% required at least one dialysis as part of treatment for HUS and the dialysis requirement was similar across the years from 28.29% in 2007 to 36.29% in 2018. 

Outcomes of HUS hospitalizations

The two major outcomes of interest in our study were in-hospital mortality and discharge to SNFs. The overall in-hospital mortality of HUS patients was 3.03%. The percentage of patients discharged to an SNF was found to be 15.69% (as compared to 81.28% who were discharged to their homes). The median LOS of hospitalizations due to HUS was eight days with an interquartile range of 3-15 days. The mean length of stay at a hospital was 12 days with a standard deviation (SD) of 0.5.

Predictors of poor outcomes of HUS hospitalizations

In line with our analysis, we have focused on two main undesirable outcomes: in-hospital mortality and discharge to SNFs. Characteristics associated with statistically significant in-hospital mortality included increasing age and dialysis requirements. Increasing age depicted 1.4 times more risk of in-hospital mortality (95%CI: 1.221-1.686; p-value <0.0001). The requirement of at least one dialysis contributed to a 2.1 times higher risk of in-patient mortality (95%CI: 1.141-4.167; p-value: <0.0001) compared to the non-necessity of dialysis in HUS patients. 

According to the study, age >65 (OR: 2.599, 95%CI: 1.406-4.803; p-value: 0.0023) had the highest odds of discharge to an SNF. Additionally, comorbidities such as diabetes mellitus and pulmonary circulatory diseases, which are under vascular events, had higher odds of discharge to an SNF (OR: 1.467, 95%CI: 1.075-2.000, p-value: 0.0156). Moreover, intravenous immunoglobulin (IVIG) and plasmapheresis had high odds of discharge to an SNF ((OR: 1.99, 95%CI: 1.307-3.03; p-value: 0.0013) and (OR: 5.509, 95%CI: 2.807-10.809; p- value<0.0001), respectively). Analysis of hospital bed sizes and hospital type were also associated with higher odd discharge to an SNF (OR: 1.849, 95%CI: 1.142-2.993; p-value: 0.0124).

## Discussion

Trends of hospitalized HUS patients

In this study, we evaluated the trends and outcomes of hospitalizations due to HUS from 2007 through 2018. Our study showed an overall decrease in hospitalizations for which HUS was the primary diagnosis whereas we saw an increase in inpatient mortality. Despite the total increase in mortality, there was a decline in the proportion of patients being discharged to skilled nursing facilities (SNFs). Further, the present study revealed that age and requirement for dialysis are associated with increased odds of mortality, which is similar to those of previous studies and provided updated trends on HUS hospitalization and in-hospital mortality in the United States.

This study showed a decline in hospitalization rate in children aged 0-10 years, while it showed a steady increase in other age groups. We have limited studies regarding the trend and outcome in hospitalized patients in the United States as well as worldwide. A previous population-based study by Noris et al., based in Italy, has documented that HUS is exceedingly rare, with an overall mean annual incidence of 6.3 cases/million children aged <18 years [3]. Post-diarrheal HUS in children is usually self-limited and only requires supportive care, with only 5% having significant sequelae as reported by a 20-year population-based study in Utah [8]. This could be the reason for the decline in hospitalization in small children. Other infectious causes also can be associated with HUS such as pneumococcal infection and a study showed that hospital discharges from 1997 to 2009 have approximately doubled [17]. Atypical HUS is a rarer presentation of this entity, which is associated with genetic dysregulation of the component pathway. In Europe, reported incidence ranged between 0.23 and 1.9 per million annually. Despite this, the overall prognosis for patients with aHUS has been poor. Initial mortality has been reported to be higher in children (6.7% versus 0.8% at one year), although adults progress to end-stage renal disease (ESRD) more frequently at initial presentation (46% versus 16%) [18]. Our study did not differentiate between typical versus atypical HUS since the genetic study is not routinely performed; however, this could be the reason for the increased hospitalization rate.

Our study also showed that in Black patients, the hospitalization trend is steadily increasing, while it is declining in other populations. It is somehow disproportionate since most hospitalized HUS patients are White. Since there is limited study pertaining to the hospitalization trend in HUS patients, the reasons are unclear. A 1994 population-based study in Washington DC showed that the incidence of HUS is low in the Black population [19]. Based on our study’s analysis of predictors of poor outcomes, however, racial predisposition is not one of them. Black populations have had an increasing trend of hospitalization for other illnesses too such as hypertension [20] and heart failure [21]. Further studies are needed to elucidate these findings.

Characteristics of hospitalized HUS patients

In our study, more than half of the cases of HUS belonged to the age group of 1-10 years (56.2%); 24.7% of patients were in the age group of 18-65 years and 9.7% of patients were in the age group of 11-17 years. This shows statistics similar to that of a study conducted on children in Italy during 2003-2012 in which 75.2% of HUS patients were less than six years old [21]. A similar study in the United States reported that 66% of HUS cases in children less than 18 years of age occurred in the less-than-five-years age group with an incidence rate of 1.9 per 100,000 children compared to an overall incidence rate of 0.78 per 100,000 children {22}. The high incidence of HUS in children less than 10 years of age as found in our study could be substantiated by the study of Slutsker et al., who found that the highest age-specific isolation proportions of *Escherichia coli* O157:H7 from fecal specimens were in the five-to-nine-years age group (0.90%) [22]. Since Shiga toxin-producing *E coli* (STEC) O157:H7 accounts for 88% of cases of pediatric HUS, it could be inferred that HUS occurred in cases with high infection rates of STEC [21].

In a population-based surveillance study of pediatric HUS patients conducted in the United States during 2000-2007, 56% of HUS cases occurred in females [23]. In a study based on a cohort of TTP-HUS patients identified through the Oklahoma Blood Institute, 77% of patients with TTP-HUS who had a bloody diarrhea prodrome were females [24]. A Norwegian retrospective study also reported that 66% of HUS in children occurred in females [25]. Clearly, there is a female predisposition to the occurrence of HUS, reiterated by our study which reported that 59.5% of patients with HUS were females. The reason for female predominance is unknown because there is no difference in the incidence of infection by STEC in males and females [23,26].

In a study conducted using the Oklahoma TTP-HUS Registry, the incidence rate of TTP-HUS was 80% in the White and 8% in the Black races. The study also reported that 97% of patients were Caucasians in the TTP-HUS preceded by a prodrome of bloody diarrhea. Our study also reported similar trends of an overall increased incidence rate of HUS in the White race (74.4%). Our study also noted an increasing incidence of HUS in the Black population in recent years with the highest incidence rate of 19.1% in 2018. In the study by Terrell et al., the relative frequency of HUS in Blacks was significantly higher in patients with a previous diagnosis of systemic lupus erythematosus (SLE) (41%) and autoimmune disease (25%) [24]. It was also found that there was a nine-fold relative increase of HUS in Blacks with ADAMTS13 (a disintegrin and metalloproteinase with a thrombospondin type 1 motif, member 13) deficiency, probably related to autoimmune etiology [26].

Our study showed that the highest percentage of HUS admissions were in the Southern region of the United States. In the study by Ong et al., the highest incidence rates were in the states of Oregon and Minnesota while Georgia, which was one of the Southern states recorded the lowest incidence. Hence, we conclude that there are no obvious geographical trends in HUS affliction [22].

In our study, 58.6% of patients were transferred from other hospitals or SNFs. In a cross-sectional study, 71.6% of patients were transferred from another SNF with reported reasons being the need for a pediatric specialist (49.1%), the need for a renal specialist (23.6%), the need for dialysis unavailable at the initial facility (23.6%), and rural or small hospital unable to provide necessary care (11.3%) [27].

Treatment of hospitalized HUS patients

Early institution of treatment does improve the prognosis of HUS. Care needs to be given to address anemia, thrombocytopenia, hypertension, and AKI.

HUS patients can become profoundly anemic. Packed RBC should be transfused whenever the hemoglobin level is < 6 g/dL and/or hematocrit level is < 18% to minimize cardiopulmonary declination. Eighty percent of children with STEC-HUS does need RBC transfusions [28]. To avoid the risk of cardiopulmonary complications with subsequent high-output heart failure, a post-transfusion goal of hemoglobin 8-9 g/dL is recommended. It is important to note that the goal isn’t to restore the hemoglobin level back to normal due to the increased risk of volume overload, which may cause hypertension, pulmonary edema, and heart failure [29].

Platelet transfusion for thrombocytopenia is rare. It’s reserved for patients with clinical bleeding or patients scheduled for a procedure. In a retrospective study, it was noted that there were no bleeding complications in 73 patients who needed invasive procedures such as central venous catheter or peritoneal dialysis catheter placement [30].

HUS patients are prone to develop high blood pressure likely due to intravascular volume expansion and/or the activation of the renin-angiotensin-aldosterone system (RAAS) secondary to ischemia. Management is aimed toward control of fluid status and administration of anti-hypertensive medications for better control and to minimize vascular events. In our study, we did conclude that there were increased vascular events in trend from 6.66% in 2007 to 9.68% in 2018.

We observed 66.9% of HUS patients who developed AKI, which showed a rise in AKI events in the last five years of our study (i.e., 77.42% in 2018 compared to 54.82% in 2007). At least one dialysis was required by 29.8% of patients as part of the treatment for HUS and the dialysis requirement was similar across the years from 28.29% in 2007 to 36.29% in 2018. Early dialysis didn’t alter the effect of clinical outcomes. Indications of dialysis in children diagnosed with HUS remain similar to the children with AKI due to the other forms, which include azotemia (defined as blood urea nitrogen (BUN) >= 80-100 mg/dL), severe electrolyte abnormalities (eg., hyperkalemia) that is refractory to medical management, uremia signs and symptoms, and volume overload (cardiac and pulmonary declination) [31].

Some patients with HUS do receive plasmapheresis. In our study, it was noted that 18.1% of patients with HUS required plasmapheresis. In addition, it was observed that treatment with plasmapheresis demonstrated a stable trend over the years from 2007 (17.76%) to 2018 (15.32%).

Outcomes and predictors of outcomes

Our studies showed increased inpatient mortality in the last decade. Advanced age and progression to needing dialysis as an inpatient are identified as statistically significant predictors of mortality. Advanced age also is identified as a predictor for discharge to facility outcome. In a French cohort study, it was demonstrated that around 60% of cases occurred in adulthood, and 98% of those patients were above the age of 65 [18]. This study showed more than half of the adult subjects developed ESKD requiring dialysis. This study also showed that without dialysis, 64% of adults relapsed, while the number is lower in children [18]. It is unclear if there is a tendency for more complement dysregulation in adults that worsen outcomes since we don’t have conclusive studies. While advanced age shows a higher OR of discharging to facilities, we also noticed a similar trend in patients with comorbidities such as diabetes mellitus and patients receiving plasmapheresis and IVIG. We could argue that patients with multiple comorbidities tend to be older and have higher frailty score and frailty is a predictor of an increased rate of discharge to facilities [32]; however, this is an inference at best and need to be proven with further studies specifically relating to HUS patients.

Limitations

There are some limitations pertaining to our study. We were only looking into hospitalization trends and, therefore, we are lacking data on long-term outcomes. In-hospital mortality data might not correlate to long-term mortality. Furthermore, since this was a retrospective study using the NIS dataset, we were not able to differentiate between typical versus atypical HUS since genetic testing was not performed. 

## Conclusions

In this population-based study, we demonstrated decreased rate of hospitalization and increased in-hospital mortality among adult hospitalizations with a primary diagnosis of HUS. There were several demographic and patient-level and hospital-level characteristics associated with in-hospital mortality and discharge to SNFs. Further surveillance is required to determine if these trends continue and future studies should focus on the reasons for the increased inpatient mortality despite the declining rate of hospitalization. An understanding of these reasons will allow for the development of interventions and strategies to sustain these trends.
